# Rates and Rocks: Strengths and Weaknesses of Molecular Dating Methods

**DOI:** 10.3389/fgene.2020.00526

**Published:** 2020-05-27

**Authors:** Stéphane Guindon

**Affiliations:** Laboratoire d'Informatique de Robotique et de Microélectronique de Montpellier, CNRS and Université Montpellier (UMR 5506), Montpellier, France

**Keywords:** fossils, calibration, Bayesian inference, relaxed clock models, fossilized-birth-death process, total-evidence, tip-dating

## Abstract

I present here an in-depth, although non-exhaustive, review of two topics in molecular dating. Clock models, which describe the evolution of the rate of evolution, are considered first. Some of the shortcomings of popular approaches—uncorrelated clock models in particular—are presented and discussed. Autocorrelated models are shown to be more reasonable from a biological perspective. Some of the most recent autocorrelated models also rely on a coherent treatment of instantaneous and average substitution rates while previous models are based on implicit approximations. Second, I provide a brief overview of the processes involved in collecting and preparing fossil data. I then review the main techniques that use this data for calibrating the molecular clock. I argue that, in its current form, the fossilized birth-death process relies on assumptions about the mechanisms underlying fossilization and the data collection process that may negatively impact the date estimates. Node-dating approaches make better use of the data available, even though they rest on paleontologists' intervention to prepare raw fossil data. Altogether, this study provides indications that may help practitioners in selecting appropriate methods for molecular dating. It will also hopefully participate in defining the contour of future methodological developments in the field.

## 1. Introduction

Telling apart the rate of molecular substitution from the time, measured in calendar units, that define periods of evolution, is the main endeavor of molecular dating techniques. The basic idea underlying these techniques is straightforward. The comparison of a set of homologous genetic sequences provides information about the number of (nucleotide, amino acid, or codon) substitutions that took place along the edges of the phylogeny connecting these sequences. If information is available about either the rate at which these substitutions take place or the actual timing of particular events in the phylogeny, then one may express the length of an edge as the product of the rate of molecular evolution by a calendar time elapsed along this edge. The application of this approach, in its simplest form (Zuckerkandl and Pauling, 1965), has led to spectacular findings—the reappraisal of the timing of divergence between African apes and humans (Sarich and Wilson, 1967) being perhaps the most emblematic. Since their first use more than five decades ago, molecular dating methods have considerably increased in sophistication. Heightened complexity indeed arose at many different levels of the analysis, going from the collection of genetic and fossil data to the reconstruction of phylogenetic trees.

This review article leaves aside many important aspects of modern techniques in molecular dating. In particular, it does not touch on the preparation of data, may it be the various algorithms for aligning homologous genetic sequences or the techniques used in the exploration of geological strata for extracting fossil data. Despite being central in Bayesian methods for the inference of node ages (see e.g., Condamine et al., 2015 for an illustration), the details of the tree-generating processes will also be largely omitted. Furthermore, computational considerations will not be discussed and I will not provide a list of available software implementing the most up-to-date techniques for molecular dating. Simulation techniques used to assess the accuracy and precision with which node ages are inferred, including the generation of phylogenies (Stadler, 2019) and genetic sequences (Fletcher and Yang, 2009; Currat et al., 2019), will also be ignored. I refer the keen reader to dos Reis et al. (2016) and Bromham et al. (2018) that give a broader overview of the various methodological and practical aspects pertaining to molecular dating.

The present work focuses instead on two specific aspects of molecular dating. It first provides an in-depth presentation of the models describing the variation of the rate of molecular evolution along a phylogenetic tree. This presentation serves as a basis to assess clock models, revealing some of the weaknesses of the most popular approaches. Note that the probabilistic models presented here focus exclusively on the evolution of the rate of substitution between nucleotides, amino-acids or codons. Yet, variations in the rate of evolution manifest themselves at other levels in molecules. For instance, the secondary structure of some proteins has been shown to evolve in a non-clock-like manner (Pascual-Garćıa et al., 2019). The mode and tempo of evolution of secondary and tertiary protein structures is beyond the scope of this study, however, and I will only deal with the evolution of primary sequences. The second part of this review deals with the techniques used for calibrating molecular dating analyses based on fossil data. Here again, statistical and biological arguments are presented that help evaluate the relevance of the main techniques, including the most recent developments such as the fossilized-birth-death model and the “total-evidence” approach.

## 2. Rates of Molecular Evolution Along Phylogenies

A substitution between two nucleotides at a particular position in a genome is the outcome of two distinct events. For this reason, it is useful to distinguish between a *mutation*, which is the outcome of a biochemical process, and a *substitution*, which involves a series of population-level events leading to the fixation of a mutation, as detailed below. The mutation that substitutes a nucleotide i∈A:={A,T,G,C} by another nucleotide *j* ≠ *i* may be modeled as a “uniformly at random” event, i.e., given *i*, all *j* ≠ *i* have the same probability of replacing *i*. It is clear however that the biological reality is more subtle than that simple model. For instance, mutations generally favor transitions over transversions. Although part of this bias is the consequence of natural selection acting on proteins, it has been shown that transitions are over-represented in pseudogenes (Gojobori et al., 1982), suggesting that the biochemical processes involved in mutations are also responsible for the observed bias. Other biochemical events, such as biased gene conversion (Duret and Galtier, 2009) for instance, invalidate to a certain extant the “uniformly at random” assumption, at least in some parts of the genomes (i.e., the regions prone to recombination) in mammals and yeast. Furthermore, mutation rates are most likely influenced by species-specific characteristics such as generation time, metabolic rate and DNA repair efficiency (Gillespie, 1994; Baer et al., 2007), such that these rates are also likely to vary extensively across lineages in the tree of life.

The second event involved in the making of a substitution is fixation. Although a mutation arises in a single genome at a given point in time, its frequency in the population, and in the species this population belongs to, may increase until it completely replaces the original allele. Note that we assume here that mutations are rare such that a mutant allele reaches fixation or vanishes from the population before a new mutation arises. The process of fixation of a mutation is complex as it is governed by various evolutionary forces such as natural selection (beneficial mutations will, on average, reach fixation more frequently and quickly than mutations leading to a decrease in fitness), genetic drift (the fixation of an allele occurs more quickly in small vs. large populations), and migrations. These three forces constitute the main focus of studies in classical population genetics and will not be discussed in more detail here. Most phylogenetic analysis techniques rely on a phenomenological approach for modeling substitutions whereby mutation and fixation are not distinguished explicitly. Note however that a substantial body of work has focused on deriving models of substitution from the basic principles of population genetics (Halpern and Bruno, 1998; Nielsen and Yang, 2003; Thorne et al., 2007; Cartwright et al., 2011).

The accumulation of substitutions between nucleotides during the course of evolution is thus generally assumed to be governed by a continuous-time Markov process. Individual sites are here considered as independent and identically distributed (iid), i.e., a simulation of the same Markov process runs along the phylogeny, at each position along the genome, to give rise to the observed data at the tips of the tree. The iid assumption is of course problematic when dealing with coding sequences. Indeed, through the action of natural selection, a non-synonymous change in a given region of the sequence may cause another non-synonymous substitution in a remote region in order to compensate for the first one. Yet, substitutions taking place at third coding positions may be considered as approximately iid and the same approximation can be made for non-coding regions of the genome or for pseudo-genes.

### 2.1. The Strict Clock Model and an Extension

At a given point in time *t* during the course of evolution, in a particular ancestral lineage labeled with index *l* and at a particular site *s*, one considers that substitutions accumulate randomly, following a Poisson point process of intensity μ(*l, t, s*). The substitution rate is generally decomposed as follows: μ(*l, t, s*) = *r*(*s*)μ(*l, t*), where *r*(*s*) describes the variability of rates across sites. This random variable generally follows a discrete gamma distribution (Yang, 1994) although the use of non-parametric distributions is now commonplace (Soubrier et al., 2012). In the following, I will ignore this extra layer in the model describing the rate at which substitutions accumulate. I will thus focus on the term μ(*l, t*) here on.

A first approach for modeling the fluctuation of the rate of substitution when considering the evolution of multiple species is to assume that μ(*l, t*) is constant throughout, i.e., μ(*l, t*) = μ. This simplification corresponds to the well-known “strict molecular clock” model pioneered by Zuckerkandl and Pauling (1965). Note that the actual (or realized) number of substitutions in a given time interval, along a particular lineage, may vary from one site to another because of the inherent stochasticity of the underlying process. Yet, these numbers of substitutions are all considered as random draws from the same Poisson distribution.

Molecular sequences can be considered as snapshots of molecular evolution at a single or a few point(s) in time. Hence, detailed information about evolutionary events at *all* points in time is forever lost and only *average* trends can be recovered from the observation. In the following, I will focus on the relationship between clock models, such as the strict clock model cited above, and average substitution rates in the context of date inference. More specifically, the (pathwise) average substitution rate, λ_*t*_, is defined as follows:

(1) $$\begin{array}{l} \lambda_{t}:=\frac{1}{t}\int_{0}^{t}\mu \left(l,x\right)\text{d}x. \end{array}$$

Hence, λ_*t*_ is proportional to the integral over the rate trajectory {μ(*l, x*), 0 ≤ *x* ≤ *t*} that gives the value of the substitution rate at all points in time in the time interval [0, *t*]. As we will see below, some clock models (uncorrelated ones in particular) focus solely on the distribution of the average rate λ_*t*_. Other approaches model instead the instantaneous rate μ(*l, x*), even though only the average can be inferred from the analysis of molecular data.

Bayesian inference of divergence times rests on the joint posterior density of model parameters given the observed data. I consider for now that data simply consists in two contemporaneous sequences, corresponding to random variables *U* and *V*, displaying sequences *u* and *v*, respectively. Beside molecular data, one also observes fossil data, noted as *I* and defining time constraints, *i*, on the age of the most recent common ancestor of the species with sequences *U* and *V*. When using a time-reversible, homogeneous and stationary Markov process describing substitutions between genetic character states, with stationary probabilities noted as π_._, the joint posterior density of interest is then expressed as follows:

(2) $$\begin{array}{l} p_{\Lambda_{t},M_{t},M_{0},T}\left(\lambda_{t},\mu_{t},\mu_{0},t|U=u,V=v,I=i\right)\\ \;\propto \mathrm{Pr}\left(U=u|V=v,\Lambda_{t}=\lambda_{t},M_{t}=\mu_{t},M_{0}=\mu_{0},T=t\right)\\ \;\times p_{\Lambda_{t}}\left(\lambda_{t}|M_{t}=\mu_{t},M_{0}=\mu_{0},T=t\right)\\ \;\times p_{M_{t}}\left(\mu_{t}|M_{0}=\mu_{0},T=t\right)\\ \;\times p_{M_{0}}\left(\mu_{0}\right)\\ \;\times p_{T}\left(t|I=i\right)\\ \;\times \pi_{v} \end{array}$$

The first term to the right of the “proportional to” (∝) sign is the probability of transitioning from state *v* (random variable: *V*) to state *u* (R.V.: *U*) along an evolutionary path that lasted *t* calendar units of times (R.V.: *T*), with instantaneous rates at the start and at the end of that path being equal to μ_0_ and μ_*t*_ respectively (R.V.: *M*_0_ resp. *M*_*t*_), and average rate (as defined in Equation 1) equal to λ_*t*_ (R.V.: Λ_*t*_).

In the expression above, the transition probability between observed characters (nucleotide or amino-acids generally) is a function of the instantaneous substitution rates at times 0 and *t*, plus the average rate in the corresponding time interval. Yet, knowing the instantaneous rates is not required. Indeed, one has:

$$\begin{array}{l} \mathrm{Pr}(U=u|V=v,\Lambda_{t}=\lambda_{t},M_{t}=\mu_{t},M_{0}=\mu_{0},T=t)\\ \;=\sum_{k=0}^{\infty }\mathrm{Pr}(U=u,N_{t}=k|V=v,\Lambda_{t}=\lambda_{t},M_{t}=\mu_{t},M_{0}=\mu_{0},T=t)\\ \;=\sum_{k=0}^{\infty }\mathrm{Pr}(U=u|V=v,N_{t}=k,T=t)\times \mathrm{Pr}(N_{t}=k|\Lambda_{t}=\lambda_{t}), \end{array}$$

where *N*_*t*_ is the random variable giving the number of substitutions that took place in [0, *t*]. The key observation here is that the distribution of *N*_*t*_ is determined by a non-homogeneous Poisson process (the parameter of this Poisson process is defined by the rate trajectory). This distribution is unaffected by the specifics of the rate trajectory. It is a function of the average rate along the trajectory only (i.e., λ_*t*_), thereby explaining why μ_*t*_ and μ_0_ vanish in the equation above. The transition probability thus simplifies to give the following expression (in a simplified notation):

(3) $$\begin{array}{l} \mathrm{Pr}\left(u|v,\lambda_{t},\mu_{t},\mu_{0},t\right)=\mathrm{Pr}\left(u|v,\lambda_{t},t\right) \end{array}$$

(4) $$\begin{array}{l} ={\left[e^{-\lambda_{t}t\text{Q}}\right]}_{v,u}, \end{array}$$

where **Q** is the generator of the Markov chain governing substitutions ([**Q**]_*v, u* ≠ *v*_ gives the rate of change from state *u* to *v*).

One may then envisage various instances of the clock model. The strict clock model corresponds to the case where the rate trajectories are deterministic such that *p*_Λ_*t*__(λ_*t*_|μ_*t*_, μ_0_)dλ_*t*_ = *p*_*M*_*t*__(μ_*t*_|μ_0_)dμ_*t*_ = *p*_*M*_0__(μ_0_)dμ_0_ = 1 when λ_*t*_ = μ_*t*_ = μ_0_ and 0 otherwise, i.e., instantaneous rates are all equal at all points in time along the considered edge (and thus equal to the average rate too). The joint probability density of the model parameters then becomes as follows:

(5) $$\begin{array}{l} p_{\Lambda_{t},M_{t},M_{0},T}\left(z,z,z,t|U=u,V=v,I=i\right)\propto {\left[e^{-zt\text{Q}}\right]}_{v,u}\\ \;\times p_{T}\left(t|I=i\right)\\ \;\times \pi_{v}. \end{array}$$

When breaking the evolutionary path between times 0 and *t* into two time intervals, [0, *s*] and [*s, t*], the product, denoted as A, of the two terms in Equation (2) describing the evolution of the rate of evolution, i.e., $A:=p_{\Lambda_{t}}\left(\lambda_{t}|\mu_{t},\mu_{0},t\right)\times p_{M_{t}}\left(\mu_{t}|\mu_{0},t\right)$ is then defined as follows:

(6) $$\begin{array}{l} A:=p_{\Lambda_{s},\Lambda_{t-s}}\left(\lambda_{t},\lambda_{t-s}|M_{t}=\mu_{t},M_{s}=\mu_{s},M_{0}=\mu_{0},S=s,T=t\right)\\ \;\times p_{M_{s}}\left(\mu_{s}|M_{0}=\mu_{0},S=s\right)\\ \;\times p_{M_{t}}\left(\mu_{t}|M_{s}=\mu_{s},S=s,T=t\right), \end{array}$$

and takes on the following value under the “standard” strict molecular clock model:

(7) $$A=\{\begin{array}{l} g_{M}(\mu )\text{ if }\lambda_{t}=\lambda_{t-s}=\mu_{t}=\mu_{s}=\mu_{0}=\mu ,\\ 0\text{ otherwise}, \end{array}$$

where *M* denotes the random variable giving the instantaneous rate of evolution everywhere in the tree under the standard strict clock model. In other words, the strict clock model assumes that average and instantaneous rates are the same everywhere in the tree, not just along individual edges as seen above.

#### 2.1.1. The “Not-so-Strict” Clock Model

Thanks to the distinction between instantaneous and average substitution rates that is made explicit here, one may design a new clock model where instantaneous substitution rates fluctuate randomly during the course of evolution but the average rate stays the same along every edge in the tree. Under the so-called “not-so-strict” clock model, the first term in Equation (6) giving the joint density of average rates along the two successive time segments, may be defined as follows:

(8) $$\begin{array}{l} p_{\Lambda_{s},\Lambda_{t-s}|\Lambda_{s}=\Lambda_{t-s}}\left(\lambda_{s},\lambda_{t-s}|\mu_{t},\mu_{s},\mu_{0},s,t\right):=\\ \;\left\{ \begin{array}{l} 0\text{ if }\lambda_{s}\neq \lambda_{t-s},\\ \frac{p_{\Lambda_{s},\Lambda_{t-s}}(\lambda_{s},\lambda_{t-s}|\mu_{t},\mu_{s},\mu_{0},s,t)}{\int p_{\Lambda_{s},\Lambda_{t-s}}(\lambda ,\lambda |\mu_{t},\mu_{s},\mu_{0},s,t)\text{d}\lambda }\text{ otherwise}. \end{array}\right. \end{array}$$

Genetic sequences combined with fossil data convey information about average, not instantaneous, rates. It is thus hopeless to try and fit the “not-so-strict” clock model to standard data sets used in molecular dating without any extra information. This model could nonetheless be relevant in particular circumstances. When considering intra-species data for instance, prior information about past variation of population sizes is sometimes available. These variations may serve as a basis to inform the part of the “not-so-strict” clock model describing the evolution of the instantaneous rates along the tree, even though the molecular clock hypothesis generally holds at that scale. More importantly, the “not-so-strict” model can be envisaged as an intermediate between relaxed and strict clock models. In case one has an informative prior about the frequency and the amplitude of the fluctuation of instantaneous rates of substitution, various molecular clock hypotheses could then be tested by comparing the strict to the “not-so-strict” models in the first place, and, depending on the outcome of that test, comparing the fit of the “not-so-strict” model to that of relaxed clock models (see next sections).

### 2.2. Uncorrelated Relaxed Clock Models

The strict and not-so-strict clock models can be expanded in two distinct ways. A first approach is to design a model whereby μ(*l, t*) = μ(*l*) for all *t* along edge *l*, i.e., each lineage has its own rate of substitution, which may differ from that of other lineages, but the rate along a given lineage *l* is constant. The rate trajectory along a branch of the phylogeny is deterministic under this model, i.e., instantaneous rates do not fluctuate randomly along each lineage. These rates can change at internal nodes in the tree though, thereby authorizing deviations from the strict molecular clock hypothesis. This model has serious conceptual issues, however, since it is not *sampling-consistent*. Sampling-consistency is a concept different from the more standard concept of statistical consistency. It was used in Barton et al. (2010) to refer to the situation where the probabilistic distribution of the age of a particular node under a given tree-generating process depends on the total number of tips in the tree. In the present context, clock models lack sampling-consistency when the number of sampled individuals (or the number of internal nodes in the tree) influences the number of potential shifts in the substitution rate. Sampling-consistency is desirable since there is no sensible reason, based on biological evidence, to believe that the rate trajectory along a particular lineage should be influenced by the number of sampled cladogenesis events taking place along it (although it may be influenced by the *total*, i.e., sampled and unsampled, lineage splits).

A second approach to define a relaxed clock model is to enforce the following constraint: $\int_{0}^{t}\mu \left(l,x\right)\text{d}x=\mu \left(l\right)t$ for all rate trajectories {μ(*l, x*), 0 ≤ *x* ≤ *t*}. In this second model, rates can fluctuate randomly along edge *l* although all the rate trajectories have to average to μ(*l*). This model is thus the “not-so-strict” equivalent to the models introduced in the previous section, in a context where rates may change across lineages. That second interpretation of the relaxed clock model is certainly more satisfactory than the previous one from a biological point of view as changes of the rate of substitution can take place at any point in time during the course of evolution. Yet, random fluctuations of the instantaneous rates naturally imply random fluctuations of the average rates too. Hence, except in the special case where the periods of time considered are very long compared to the time scale at which the instantaneous rate varies noticeably (in which case the average rates taken over multiple trajectories should all converge to a fixed value) there is no strong reason to believe that the average rate along a given edge is not a randomly varying quantity. As will be seen below, alternative models exist that are more realistic than the relaxed clock ones, without compromising on the computational burden involved.

A first relaxed clock model assumes that the average rate of substitution along a branch is exponentially distributed (Drummond et al., 2006). This model is a popular choice as it is implemented in the BEAST 1 (Drummond and Rambaut, 2007) and BEAST 2 (Bouckaert et al., 2014) packages. According to it, μ(*l*) is the realization of a random variable exponentially distributed with parameter 1/μ. The exponential model is therefore a clock model as all lineages are governed by the same parametric distribution. It is *relaxed* since the average rate along each branch is taken as a new random draw from this distribution.

The exponential relaxed clock model considers that the average rate of substitution along a given branch has a mean equal to μ and a variance equal to μ^2^. One thus relies here on a model in which the larger the average substitution rate, the larger its variance, which is consistent with the idea that large quantities vary more than smaller ones. Perhaps surprisingly, a more detailed analysis of this model reveals that the extent of deviation from the strict clock constraint does *not* depend on how fast (or slow) substitutions accumulate. For instance, in the case where μ = 1 substitution per unit of time, the probability for a lineage to evolve twice as fast as μ (or faster) is 0.14. When μ = 0.1, this probability is also equal to 0.14. This property of the exponential distribution is reflected in the excess kurtosis, which measures how likely it is to observe extreme values in a probabilistic distribution. For the exponential family, the kurtosis is not a function of the mean or the variance (it is in fact simply equal to six). Hence, it is counter-intuitive that large deviations from the strict clock have the same probability to occur when molecules evolve slow or fast, even though the variance of the average rate is larger when sequences evolve quickly. More importantly, molecules that evolve slowly may be doing so as a consequence of stabilizing selection. In this case, natural selection may prohibit large deviations from the strict clock, and doubling the rate at which substitutions accumulate may be very unlikely indeed. In a symmetric manner, neutrally evolving sequences may have more latitude to double (or halve) their rates of evolution in the real world. In summary, the exponential distribution model may provide a reasonably good approximation of the true underlying distribution only for fast- *or* slow-evolving sequences, but not for both of them.

Beside the exponential family, the fluctuations of average rates of evolution across edges in the tree are often described by a lognormal or a gamma distribution (Lepage et al., 2007). Both models are available in BEAST 1 and 2 as well as in MrBayes 3.2 (Ronquist et al., 2012b). While the exponential distribution is fully specified with just one parameter, the lognormal and gamma distributions rely on two parameters instead. These two parameters set the mean and the variance of the corresponding distributions in a separate manner, i.e., without any “hard-coded” constraints as for the exponential family that impose a quadratic relationship between the mean and the variance. Moreover, the statistical properties of the lognormal and the gamma distributions are such that slow-evolving sequences are less likely than fast-evolving ones to deviate strongly from the strict clock constraint. The lognormal and gamma families thus appear superior to the exponential distribution from a biological point of view, although the increased realism comes at the cost of estimating an extra parameter.

Beside considerations regarding the properties of various relaxed clock models when focusing on a single edge of the tree, important observations are to be made when expanding our focus to whole evolutionary paths between the root and the tips of the phylogeny. Under the uncorrelated clock models, the average substitution rate along edges in the phylogeny are all iid random variables. The sum of edge lengths on a path between the root and a tip of the tree is thus a weighted sum of iid random variables, the weights corresponding to the times elapsed along every edge on that path. Assuming that these weights are all equal to one, the sum of *k* average rates along a path is given by $Z_{k}:=\overset{k}{\underset{i=1}{\sum }}\mu \left(i,1\right)$. Invoking the central limit theorem, the random variable *Z*_*k*_ is thus asymptotically normally distributed (i.e., when *k* → ∞) with mean μ*k* and variance σ^2^*k*, where μ and σ^2^ are the expectation and the variance of μ(*i*, 1) respectively. Hence, in layman's terms, tip to root distances fluctuate more in large trees (large in terms of the number of tips, *k*) compared to small ones. This behavior is difficult to justify from a biological perspective as one would expect the patterns of rate variation along a single lineage to be unaffected by the total number of lineages included in the sample. All uncorrelated clock models have the same behavior in that regard. Moreover, this unwelcome relationship between variance and number of tips is likely to impact phylogenies in a differential manner depending on their shape, with comb-like phylogenies showing highly variable numbers of edges between the root and tip nodes, while more balanced tree shapes show less extreme variation in the length of these paths. In that regard, selecting subsets of taxa so as to make the phylogeny more balanced probably helps circumventing this issue. Yet, this practice may lead to increasing sampling errors due to the decreasing amounts of data available for the dating analysis.

I have assumed that the time elapsed along each branch was equal to one in the previous paragraph. I also focused on the distribution of a single tip-to-root distance. In practice, however, amounts of time elapsed along branches of actual phylogenies vary between edges. Moreover, one is usually interested in the variance of tip-to-root distances *within* a tree, not *across* trees. This “within-tree” variance is harder to characterize analytically due to the correlation between tip-to-root paths as defined by the tree. I thus performed simulations where trees were first generated according to a birth-death process with sampling. The TreeSim package (Stadler, 2019) available in the R programming language was used here in order to simulate trees conditioned on a given number of sampled tips. The birth and death rates were set to 1.0 and 0.5, respectively and 20,000 trees were generated. Each tree was obtained by simulation of a birth-death process with 100 species. The fraction of sampled taxa was chosen uniformly at random in [0.1, 1]. The length of each edge in these birth-death trees was then multiplied by a random draw taken from an exponential distribution with the rate set to 1.0. Tip-to-root distances were extracted from each tree using the phytools package (Revell, 2018) and their variance calculated.

**Figure 1:**
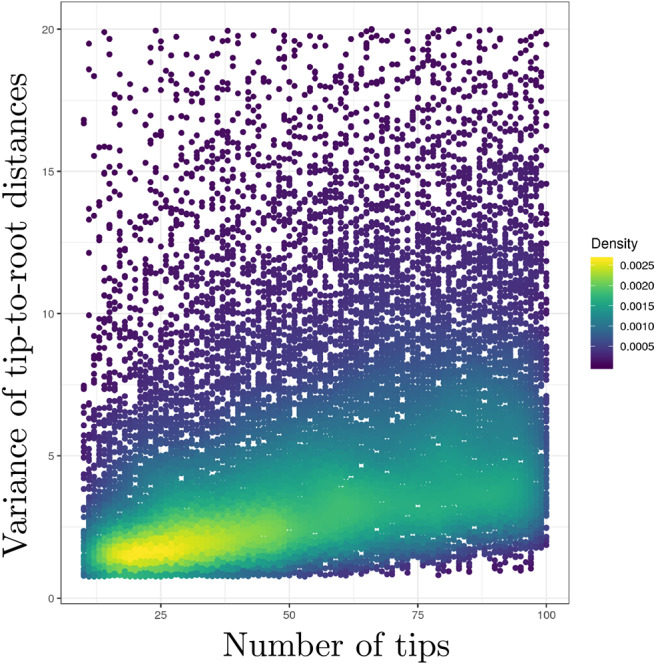
Uncorrelated clock models produce stronger deviations from the strict clock constraint in larger trees. *y*-axis: variance of the tip-to-root distances. *x*-axis: number of tips. Deviation from the strict molecular clock hypothesis was generated using the uncorrelated exponential model. The scatterplot derives from the analysis of 20,000 observations, each observation being a value for the tip-to-root variance and the corresponding number of tips.

Figure 1 gives the plot of the number of tips (on the *x*-axis) against the variance of the tip-to-root distances in each tree (*y*-axis). This plot confirms the positive correlation between the number of tips in the tree and the variance of the tip-to-root distances. Similar results were obtained with the gamma model (see Figure S1) while the Kishino et al. (2001) autocorrelated clock model does not display obvious signs of positive correlation (Figure S2). In the context of the Bayesian inference of divergence dates, uncorrelated clock models therefore define prior distributions on rates of evolution that entail stronger deviations from the strict clock in larger trees. This behavior may impact the inference of node ages. Indeed, an increased variability in rates along lineages in data sets with large numbers of tips may be responsible for an inflation of the variance in the age estimates themselves. Note also that this inflation may preferentially impact the ages of nodes along lineages that are composed of a large number of branches compared to those consisting of only a few branches. Although simulation studies generally focus on the accuracy with which ages are estimated, considering the impact of the various models of rate variation on the precision of these estimates should therefore be examined too.

Furthermore, Lepage et al. (2007) mention that defining a model where rate *trajectories* along each edge define gamma- or exponentially distributed *average* rates is not trivial (the same argument applies to the uncorrelated log-normal model). They state that these “trajectory models” would display rate autocorrelation within each edge, even though the trajectories across distinct edges would be truly independent. Hence, here again, the uncorrelated clock models appear to be sampling-inconsistent: the amount of (instantaneous) rate autocorrelation depends on the number of internal nodes (and thus the number of tips) in the tree. Heath et al. (2012) describe a more sophisticated uncorrelated clock model whereby the average substitution rate along each branch derives from a Dirichlet process prior (DPP). This model assumes a finite number of rate values. Each of these rates is considered as a random draw from a gamma distribution whose parameters are fixed *a priori*. The number of rate classes is estimated from the data through a so-called “concentration” parameter. The DPP model is thus conceptually very close to a slightly modified version of the standard uncorrelated clock model where rates follow a discretized gamma distribution. Further investigations would be required though in order to assess whether the DPP model suffers from the same shortcomings as that discussed above. It seems likely however that all uncorrelated models proposed so far, including DPP, lack sampling-consistency and implicitly rely on the questionable assumption that large trees (in terms of their number of tips) deviate more from the strict clock hypothesis than smaller ones.

### 2.3. Autocorrelated Clock Models

Equation (6) gives a generic expression characterizing the distribution of the instantaneous rates at three successive points in time along with that of the corresponding average rates. It is relatively straightforward to introduce correlation between substitution rates along the tree in this framework. The first model explicitly accommodating for autocorrelation was proposed by Thorne et al. (1998). Instead of modeling instantaneous rates at times *s* and *t*, corresponding to the end nodes of the first and second edge respectively, they focused on the rates at times *s*′: = *s*/2 and *t*′: = (*t* + *s*)/2, i.e., the “middle” of the corresponding two branches. Equation 6 therefore needs a slight re-writing to yield:

(9) $$\begin{array}{l} A:=p_{\Lambda_{s},\Lambda_{t-s}}\left(\lambda_{s},\lambda_{t-s}|M_{t^{\prime }}=\mu_{t^{\prime }},M_{s^{\prime }}=\mu_{s^{\prime }},S=s,T=t\right)\\ \;\times p_{M_{t^{\prime }}}\left(\mu_{t^{\prime }}|M_{s^{\prime }}=\mu_{s^{\prime }},S=s,T=t\right)\\ \;\times p_{M_{s^{\prime }}}\left(\mu_{s^{\prime }}|S=s\right). \end{array}$$

The instantaneous rate transition probability density (i.e., $p_{M_{t^{\prime }}}\left(\mu_{t^{\prime }}|M_{s^{\prime }}=\mu_{s^{\prime }},S=s,T=t\right)$) is given by a lognormal distribution with $\text{E}\left[M_{t^{\prime }}^{n}\right]=e^{n\log\left(\mu_{s^{\prime }}\right)+n^{2}\sigma^{2}\left(t^{\prime }-s^{\prime }\right)/2}$. The first two moments fully specify the whole distribution. One thus assumes here that the logarithm of the instantaneous rate at time *t*′ is a normally distributed random variable with a mean equal to the logarithm of the instantaneous rate at time *s*′ and variance proportional to *t*′ − *s*′. The mean of the lognormal distribution is thus equal to $\mu_{s^{\prime }}\times e^{\sigma^{2}\left(t^{\prime }-s^{\prime }\right)/2}$, which is larger than the ancestral rate $\mu_{s^{\prime }}$ in general, thereby leading to an unwarranted increase of the substitution rate over time. Kishino et al. (2001) fixed this issue by replacing the original normal distribution with one with a mean of $\log\left(\mu_{s^{\prime }}\right)-\sigma^{2}\left(t^{\prime }-s^{\prime }\right)/2$ so that the mean of the lognormal distribution is now equal to $\mu_{s^{\prime }}$, the ancestral rate.

**Figure 2:**
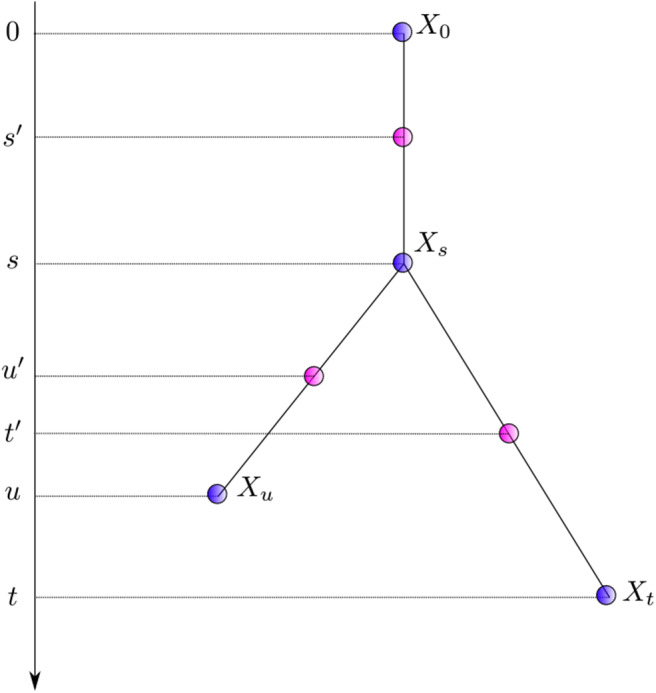
Notation for nodes and times. *X*_*i*_ denotes a node with time *t*_*i*_. Times of nodes (in purple) and that corresponding to the middle of each edge (in pink) are presented to the left.

The autocorrelated lognormal model assumes that the logarithm of the instantaneous rate follows a Brownian process. The rate itself thus evolves according to a geometric Brownian process. This model captures the idea that instantaneous rates of evolution vary little over short periods of time while longer time intervals may bear stronger fluctuations. The amplitude of these fluctuations is governed by the parameter σ which may be estimated from the data. This model also introduces correlation of rates between sister lineages. Indeed, when considering sister edges ending with nodes *X*_*t*_ and *X*_*u*_ in Figure 2, the random variables $M_{t^{\prime }}$ and $M_{u^{\prime }}$ are not conditionally independent given $M_{s^{\prime }}$ since both of them share the evolutionary path between *s′* and *s*. In practice, the non-independence between sister edges seems to have been disregarded so that only an approximation of the joint density of instantaneous rates was used instead. Kishino et al. (2001) acknowledged this problem, later proposing a different model that corresponds to that defined by Equation (6), i.e., *M*_*s*_ and *M*_*t*_, the rates at the end of the corresponding edges, replace the mid-point rates $M_{s}^{\prime }$ and $M_{t}^{\prime }$.

Although the autocorrelated log-normal model provides what can be considered a reasonable description of the evolution of the instantaneous rates along a phylogeny, Lepage et al. (2006) point out that it does not agree with some of the tenets of evolutionary biology. Indeed, the theory of episodic evolution, where periods of adaptation are followed by evolutionary stasis, implies that high evolutionary rates are more likely to decrease than the contrary. The geometric Brownian assumes instead that, at a particular point in time, the substitution rate is as likely to double as it is to halve. Lepage et al. (2006) also convincingly argue that the distribution of the rate at time *t* = +∞ should be unique, i.e., it should not depend on what the rate is at time *s* < *t*, which is not the case for the geometric Brownian process.

The Ornstein-Uhlenbeck (OU) model is a diffusion process that, unlike the geometric Brownian, satisfies this last property. However, it can take on negative values, which is not relevant when modeling rates of evolution. Aris-Brosou and Yang (2002) used the OU process in a Bayesian molecular dating approach nonetheless. It is not clear how the constraint of non-negativity of rates was implemented in this study, however. Lepage et al. (2006) proposed to use the Cox-Ingersoll-Ross process (Cox et al., 2005) instead. This process is a generalization of the squared OU model. It thus describes the random fluctuations of non-negative quantities. As for the OU model, the CIR process also has a unique limiting distribution. Two parameters specify the variance and the autocorrelation in rate trajectories in an independent manner. A third parameter defines the mean of the limiting distribution.

Beside the theoretical properties of the various clock models, practical aspects should also be considered carefully—the most important one being perhaps the relevance of the various models in the context of data analysis. Using simulations, Ho et al. (2015a) showed that detecting autocorrelation between rates is difficult so that uncorrelated and autocorrelated models often provide equally good fits to the data. Analyzing a large primate data set, dos Reis et al. (2018) observed however that the choice of rate model (autocorrelated vs. uncorrelated) has a substantial impact on the date estimates. An autocorrelated rate model provides here a significantly better fit than the uncorrelated model tested in their study. Even though autocorrelated rates do not always outperform uncorrelated ones, using autocorrelated rate models in cases rates are in fact not correlated should not, at least in principle, lead to poor date estimates. Hence, as long as the uncertainty around rate autocorrelation is taken into account in the inference, using autocorrelated clock models in practice seems preferable.

It is also essential to take into account what is known about the biology of substitution rate evolution when comparing the merits of various modeling approaches. Effective population size is one of the factors regulating the rate at which substitutions accumulate through its impact on the strength of genetic drift and selection. It is not clear however whether variations in population size during the course of evolution follow uncorrelated or correlated patterns (Lanfear et al., 2014). Life history traits such as body mass, body size and temperature, metabolic rate and generation time are also associated with the substitution rate (Levy Karin et al., 2017). Body size has been modeled as a diffusion process (see e.g., Clauset and Erwin, 2008), resulting in certain degree of autocorrelation. Yet, just because body size evolves in an autocorrelated fashion does not imply that substitution rates should follow the same patterns. It is thus safe to assume that population size and life history traits probably evolve in a seemingly uncorrelated manner when considering only distant species, so that uncorrelated models of substitution rate are appropriate at that scale. When the analysis focuses instead on closely related species and shorter evolutionary time scales, autocorrelated models are probably more relevant.

Furthermore, all models discussed here are clock models. They all assume that instantaneous substitution rates fluctuate around some average in an autocorrelated or uncorrelated manner. The fact that this average is shared by all lineages makes these models clock-like. It may be relevant to deviate from the clock assumption in particular circumstances though. Specifying multiple independent clock models may indeed be pertinent in cases where the biology of a subset of organisms is markedly distinct from that of the other taxa analyzed. For instance, comparative analyses including prokaryotes and organelles (Esser et al., 2004) may require two distinct clocks. In that respect, the “random local clock” model proposed by Drummond and Suchard (2010) addresses this particular need even though, strictly speaking, lineages still evolve under a clock model here.

#### 2.3.1. Rate Trajectories vs. Averages

**Figure 3:**
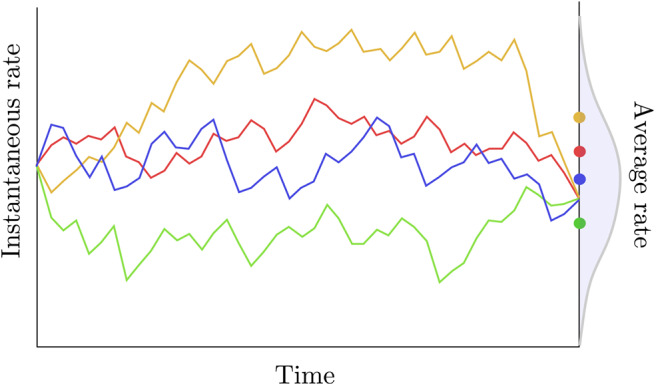
Rate trajectories and average rates. The colored lines describe rate trajectories, i.e., values of the instantaneous rate of substitution at all points in time. The four trajectories have the same instantaneous rate at the beginning and at the end of the time period considered here. Traditional autocorrelated clock models would thus assign the same length to the corresponding edge in all four cases here. The dots to the right give the average rate for each trajectory taken over that same time period. These averages are iid random quantities whose distribution is given in gray.

Going back to Equation (6), the question of defining the probability density of the mean rate along a given branch given the instantaneous rates at its extremities remains. Kishino et al. (2001) rely on a strong assumption about the corresponding distribution. Indeed, the corresponding probability density is defined as follows:

(10) $$\begin{array}{l} p_{\Lambda_{s}}\left(\lambda_{s}|M_{s}=\mu_{s},M_{0}=\mu_{0},S=s\right)\text{d}\lambda_{s}:=\\ \;\{\begin{array}{ll} 0 & \text{if }\lambda_{s}\neq (\mu_{0}+\mu_{s})/2\\ 1 & \text{otherwise}, \end{array} \end{array}$$

i.e., the distribution of the average rate has a point mass probability set at the average of the instantaneous rates at the two extremities of the edge considered. Two observations can be made regarding this definition. First, assuming that the rate trajectories are governed by a geometric Brownian process with a small variance parameter (σ) and/or considering a short period of time, then the trajectories are approximately linear and the average rate is indeed equal to the arithmetic mean of the instantaneous rates at the beginning and at the end of each trajectory. Kishino et al. (2001) also assume that the variance of the average rate is null. Here again, this assumption is only reasonable in the particular case where σ is small and/or a short time interval is considered. In general, however, given the instantaneous rates at both extremities of an edge and assuming geometric Brownian trajectories between these two values, the average rate along the branch should be treated as a random variable with potentially non-zero variance (Figure 3).

Lepage et al. (2006) were the first to clearly point out that assuming random trajectories for instantaneous rates implies that the average rate along each edge should also be considered random. Acknowledging the randomness of the average substitution rate poses the question of the derivation of transition probabilities between character states along edges of the phylogeny. Replacing the expression for the transition probability in Equation (2) by that given in Equation (4) yields:

(11) $$\begin{array}{l} p_{\Lambda_{t},M_{t},M_{0},T}(\lambda_{t},\mu_{t},\mu_{0},t|U=u,V=v,I=i)\propto {\left[e^{-\lambda_{t}t\text{Q}}\right]}_{u,v}\\ \;\times p_{\Lambda_{t}}(\lambda_{t}|M_{t}=\mu_{t},M_{0}=\mu_{0},T=t)\\ \;\times p_{M_{t}}(\mu_{t}|M_{0}=\mu_{0},T=t)\\ \;\times p_{M_{0}}(\mu_{0})\\ \;\times p_{T|I}(t|i)\\ \;\times \pi_{v}. \end{array}$$

This last expression suggests that Bayesian inference of divergence dates should incorporate instantaneous rates at all nodes in the tree plus the corresponding average rate along each edge as latent variables, which could be effectively “integrated over” using standard Metropolis-Hasting operators for instance. Lepage et al. (2006), Guindon (2012), and Privault and Guindon (2015) went one step further by showing that it is in fact possible to drop the average rates from the (rather long) list of latent variables. The posterior distribution of interest then becomes:

(12) $$\begin{array}{l} p_{M_{t},M_{0},T}(\mu_{t},\mu_{0},t|U=u,V=v,I=i)\propto \int {\left[e^{-zt\text{Q}}\right]}_{v,u}\\ \;\times p_{\Lambda_{t}}(\lambda_{t}|M_{t}=\mu_{t},M_{0}=\mu_{0},T=t)\text{d}z\\ \;\times p_{M_{t}}(\mu_{t}|M_{0}=\mu_{0},T=t)\\ \;\times p_{M_{0}}(\mu_{0})\\ \;\times p_{T|I}(t|i)\\ \;\times \pi_{v}. \end{array}$$

The transition probability is thus derived here by integrating over all possible values that the average rate can take conditioned on the instantaneous rates at the branch extremities. The corresponding integral (i.e., the first term to the right of the equation above) can be solved analytically, or approximated, in some circumstances. I refer to this calculation as the “integrated average-rate approach,” or IARA, in the following. Lepage et al. (2006) provide a closed-form formula for a IARA assuming that the instantaneous rates evolve under the CIR process. They used a simplified version of it in order to evaluate the likelihood on a three-taxon star-like tree. In a subsequent study, Lepage et al. (2007) used the mean of the distribution of the average rate under the CIR but assumed a null variance. In Guindon (2012), I focused instead on the geometric Brownian process, providing an approximation for the distribution of the average rate. The calculation of the transition probabilities under this IARA entails the same computational cost as that spent when considering that the average rate is not random. Privault and Guindon (2015) later examined this approximation further, confirming its validity for realistic ranges of parameters. They also provide a closed-form formula for the transition probabilities, although numerical precision issues may hamper the calculation in particular circumstances.

Another model describing the evolution of the rate of evolution is the compound Poisson process proposed by Huelsenbeck et al. (2000). This model properly accommodates the randomness of the average rate along edges in the phylogeny. However, it relies on augmenting the data by assuming that the instantaneous rate is known at every point in time, along all lineages in the phylogeny, making this approach computationally expensive. The CIR and geometric Brownian IARAs thereby appear as the best options available so far to fully accommodate for the random fluctuations of the average rate of substitutions along edges due to the underlying stochastic process governing the instantaneous rate of evolution. Beyond their ability to describe rate trajectories in a sound mathematical framework, considering the variation of the average rate along each edge offers flexibility in accommodating for site-specific processes. Indeed, these approaches take into account the site-specific variation of the substitution rate along lineages in the very same way the covarion model (Fitch and Markowitz, 1970; Tuffley and Steel, 1998) does, i.e., by authorizing each site and each edge to evolve under its own rate of evolution. Despite these obvious advantages compared to alternative models, IARAs have not been used widely so far, most likely because of a lack of implementation in popular phylogenetics software (although the geometric Brownian model is implemented in PhyTime, a software program that is part of the PhyML package).

## 3. Calibrating the Clock

Molecular dating goes beyond standard phylogenetic reconstruction by separating rates of molecular evolution from actual (i.e., calendar) times. Separating rates and times requires additional data in order to calibrate the estimated trees. Extra data may take three distinct forms: (1) information about the substitution rate, (2) information about paleogeography, or (3) fossil data.

### 3.1. Dating Without Fossils

Although data about the mutation rates in model organisms is available (see e.g., Drake et al., 1998), information about substitution rates is relatively scarce. The only noticeable exception concerns fast-evolving viruses. The pace at which some viruses (HIV or influenza for instance) evolve indeed makes it possible to obtain sequence data at different points in time such that non-negligible numbers of substitutions have accumulated between these time points (see Shankarappa et al., 1999; Biek et al., 2015, and the excellent internet resource https://nextstrain.org/, Hadfield et al., 2018). It then becomes feasible to infer a “global” substitution rate, and models of (relative) rates of evolution such as those introduced previously describe fluctuations around this trend. I will not elaborate further on serially sampled data for molecular dating here.

Information on the timing of past evolutionary events may also be informed by knowledge about geological events such as the appearance of land bridges or the emergence of islands (Heads, 2005). Indeed, assuming that geography drives speciation [through vicariance, geodispersal, or biological dispersal (Ho et al., 2015b)], the age of an island may, in some circumstances, provide a maximum (i.e., older) age for the birth of ancestral species that colonized this territory. In a symmetric manner, the appearance of land bridges is a necessary condition to explain speciation by vicariance for some species, here again potentially defining a maximum age for some internal nodes in the reconstructed phylogeny. The same land bridges can create barriers of dispersal (e.g., the Isthmus of Panama, that arose 3.5 Mya, is a barrier of migration between the Atlantic and Pacific oceans), thereby providing minimum rather than maximum ages for particular speciation events. Under this line of reasoning, one expects to observe a correlation between the splits corresponding to cladogeneses in a phylogeny and important geological events, mostly involving breakup sequences of Gondwana and Laurasia (Croizat, 1962). Yet, evidence for such correlation is difficult to ascertain (Heads, 2005) and there are examples where important geological events and cladogeneses appear to be disconnected. Hence, many terrestrial animals display strong capability for overseas dispersal so that the appearance of land bridges cannot always be used to establish a maximum age (see de Queiroz, 2005 for a review). Nonetheless, the rising and disappearance of physical connections between geographical regions on the globe is likely to influence the speciation process. Rigorous mathematical modeling, such as that proposed by Landis (2017) for instance, should thus be considered as an important step forward in molecular dating analyses and more generally in ecology.

### 3.2. Dating With Fossils

#### 3.2.1. Pre-processing of Fossil Data

Fossil data is another source of information commonly used for molecular dating. It consists in a fairly intricate combination of time and morphological information. Time information is only indirect. It is derived from the estimated age of the sediments in which the extinct taxon was collected. The age of these sediments is itself often derived indirectly from that of rock bodies that “bracket” the sediments of interest (Sterli et al., 2013). Moreover, little information is available about the way the different stratigraphic ranges were sampled in general. The data produced by paleontologists generally consist in the combination of a fossil description and the stratigraphic layer in which this fossil was found. Additional information about the experimental design, including the number and types of geological layers surveyed or the excavation techniques that were used, is often difficult to access. That lack of information is problematic. For instance, from a mathematical modeling perspective, finding a particular fossil after searching a single stratigraphic layer is very different from finding the same fossil after examining multiple layers. Finally, the rock record itself is highly heterogeneous in space because of plate tectonics and net erosion, thereby complicating even further the task of finding and interpreting fossil data (Benton et al., 2009).

Morphological information is also difficult to deal with. First, the analysis of one or multiple specimens of a given fossil taxon by paleontologists leads to a selection of “informative characters.” These morphological characters are selected based on the phylogenetic signal they convey and result from complex taphonomic processes. Characters showing apomorphies are selected. These characters show evidence of derived states in a subset of extant and extinct species while other species display ancestral states for the same character. The subset of species showing apomorphies may vary from one morphological character to another. For instance, a given fossil may display a particular character state that is shared by species A and B but not by species C and D. This fossil may also display a second character with derived states shared by A, B, and C but not by D. Both morphological characters point to different phylogenetic placements for the fossilized species, thereby generating *de facto* uncertainty in the calibration of the time tree, even though the phylogenetic relationships between A, B, C, and D may be known with good precision. The fact that the selection of a subset of morphological characters is not random (i.e., characters are selected based on their variability across sampled species) may also be considered problematic (see section 3.2.3). Finally, it is commonplace to assemble morphological characters from multiple specimens of fossils that are deemed to belong to the same ancient species (Parham et al., 2011). Here again, this step relies on the interpretation of evidence by paleontologists. In this context, it is important to stress that the validity of molecular dating analyses relies heavily on the ease with which the whole scientific community can access raw fossil data along with detailed information about how this data was processed in order to define calibration constraints. Without open and systematic access to well-curated and extensive databases of raw fossil data, dating experiments will not be fully reproducible, thereby harming our field of research. Fortunately, rich sources of information about the way fossil data is prepared prior to dating analysis can be found online. For instance, the Fossil Calibration Database^1^ (Ksepka et al., 2015) set out to use the rigorous set of guidelines defined in Parham et al. (2011). It provides useful, if partly outdated, information to generate calibration constraints for more than 200 clades and does so on a transparent forum that is open to the whole scientific community. Note however that knowledge about fossil data is constantly evolving (see e.g., Marjanović, 2019) and databases such as the Fossil Calibration Database require ongoing and constant efforts in order to remain relevant.

#### 3.2.2. Expert-Based Analysis of Fossil Data

A fossil provides a minimum age for the smallest extant clade to which it belongs, i.e., the youngest node from which it as well as any two or more extant taxa are descended. The phylogenetic position of a fossil is determined either by a phylogenetic analysis of (a subset of) its parsimony-informative characters or by a manual comparison with a list of apomorphies derived from a prior phylogenetic analysis or from prephylogenetic taxonomic work. Both approaches leave varying amounts of uncertainty, depending in part on how fragmentary the fossil in question is. Every fossil-based calibration thus contains age uncertainty and phylogenetic uncertainty.

Fossils that branch near the tips in the calibrated clade define looser younger ages for that clade compared to older fossils that branch closer to the basal node. As a consequence, paleontologists are always eager to discover older fossils that still belong to the clade of interest. Unfortunately, older fossils did not have sufficient time to accumulate as many apomorphies as younger fossils did. Early members of a given taxon were also likely to be scarce and occupy a limited geographic area (Marshall, 2019). As a consequence, older fossils are also the most difficult to associate to well-defined clades. Considerable uncertainty into the placement of these fossils in the phylogenetic tree may then hamper further analysis. At the other extremity, young fossils are likely to sit at the end of long branches, along which numerous morphological changes accumulated that took place along this branch only, potentially leading to a saturation of the signal.

Linking a given fossil to a group of taxa as is done here involves a considerable amount of work and discussions among multiple experts, as demonstrated by the wealth of information provided in the journal Palaeontologia Electronica for animals, for instance (see https://palaeo-electronica.org/content/fc-1). Note however that only taxa considered as important receive high levels of scrutiny. Hence, existing databases can be used to find well-curated calibration information for “popular” taxa, in which cases researchers rely on previous rigorous work by paleontologists in order to calibrate their own analysis. Calibrating an analysis of less well-characterized taxa generally involves a thorough search of the primary literature followed by an in-depth comparative analysis of raw morphological data as briefly explained in the previous paragraphs (see also section 3.2.1 and Saladin et al., 2017 for an example).

##### 3.2.2.1. The fossilized-birth-death model and other model-based approaches to calibration

As seen above, defining younger bounds for calibration intervals is difficult, although the fossil record provides rich information to conduct this task in some cases. Deriving older bounds is even more challenging. Indeed, the younger bound for a given clade does not put any constraint on the older bound for a nested clade. In other words, the younger bound for the MRCA of a clade made of species A, B and C does not convey any relevant information about the older bound for the MRCA of the subclade made of A and B. Beside the intervention of paleontologists, probabilistic modeling can also be used to define older calibration bounds. The two techniques described below rely on mathematical models depicting the processes governing fossilization in order to define older bounds of calibration constraints whose position in the tree is defined as outlined above.

Tavaré et al. (2002) developed a probabilistic model and an inference technique to estimate the length of the temporal gap between the oldest fossil and the time of the MRCA of a given clade, along with a confidence interval for that length. This method uses as input the number of extant species in the clade whose age is to be calibrated, the ages of the relevant stratigraphic layers, the number of fossils found in them and the (relative) fossil sampling intensities in these layers. Although this approach relies on a sound mathematical model of species diversification and properly accommodates for the specifics of the collection of fossil data, it has not been used widely so far, most likely because of the lack of software implementing it and, perhaps, the lack of information regarding fossil sampling intensities. In a very thorough study about the treatment of fossil data to calibrate the molecular clock, Marshall (2019) also described a method similar to that of Tavaré et al. (2002), where the time elapsed between the divergence date to be calibrated and the age of the oldest fossil of a given focal clade has a probabilistic distribution whose parameters depend on the number of fossil localities for that clade.

**Figure 4:**
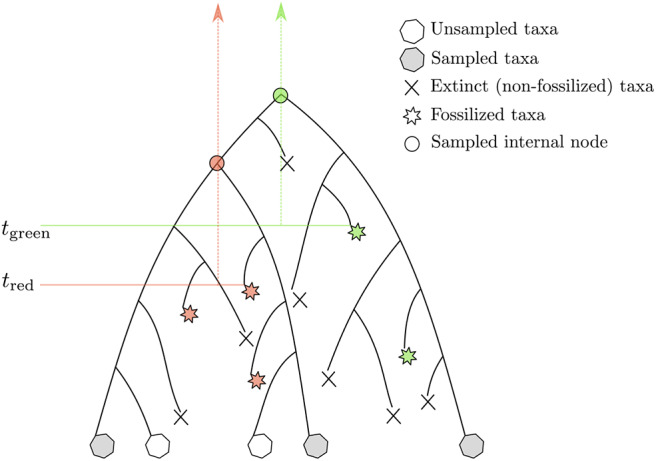
Rationale underlying the calibration of the molecular clock with the FBD model. The stars point to fossils. The fossils in red (green) calibrate the internal nodes presented as disks of the same color. The vertical arrows define the corresponding calibration intervals. *t*_red_ and *t*_green_ refer to the lower bounds associated to these two intervals. The corresponding upper bounds are determined by the birth, death, and fossilization parameters of the FBD model.

Another attempt to tackle the same issue was proposed by Stadler (2010) and Didier et al. (2012). The proposed approach is based on a probabilistic model describing the tree-generating process. Yet, it still relies on expert knowledge for placing calibration constraints in a tree. The model put forward in these two studies lies now at the core of the so-called “tip-dating” methods whereby calibrating the molecular clock derives from time information available at the tips of the phylogeny corresponding to both extant and extinct (and fossilized) species. Both approaches model the stochastic process generating a tree including sampled extant and fossil species. The so-called fossilized birth-death process (FBD) assumes that lineages give birth to new species or die at given per capita rates, which are deemed to be constant during the course of evolution. An ancestral lineage may also fossilize, an event that takes place at a particular rate, which is to be estimated from the data. After sampling, only a subset of extant and extinct species are available for the analysis (Figure 4). The joint probability density of the age of “observed” lineage splits in a phylogeny given the time of sampling of extant and extinct taxa, along with the birth, death, fossilization rates, and sampling fraction, can be evaluated analytically. When considering the calibration of one particular node using the FBD model, one has to truncate the joint probability density of node ages such that its value is null whenever the node is younger than the oldest fossil used for this particular calibration. This truncation is fairly straightforward to deal with in the context of Bayesian molecular dating using Markov Chain Monte Carlo techniques, and Heath et al. (2014) provide an analytical solution to this problem. Younger bounds for calibration intervals thus derive directly from the analysis of the fossil data. Older bounds are defined indirectly and depend on the values of the FBD model parameters. More specifically, information about the relative node heights derives mainly from the analysis of genetic sequences only (i.e., calibration data is less informative about the phylogeny itself). These relative heights then serve as a basis to infer the parameters of the FBD model which, in turn, help defining older bounds for the ages of the calibrated nodes. One thus relies here on a hierarchical model whereby the phylogeny is treated alternatively as a parameter and as data, depending on the level in the hierarchy that is considered.

##### 3.2.2.2. Performance of model-based approaches for defining calibration constraints

**Figure 5:**
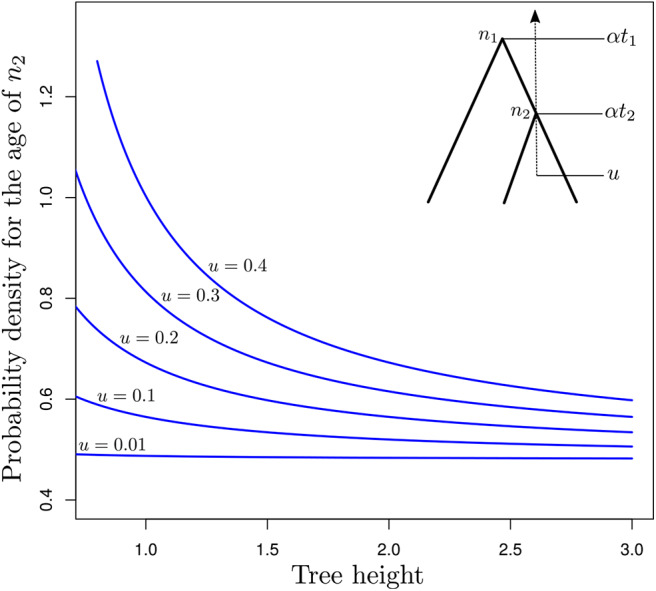
Probability density of the age of *n*_2_ as a function of the tree length for various calibration constraints. The y-axis gives $p_{\text{BD}}\left(\alpha t_{2}|\alpha t_{1},\alpha t_{2}\geq u,\frac{\lambda }{\alpha },\frac{\mu }{\alpha }\right)\text{d}\left(\alpha t_{2}\right)$ as a function of α (x-axis) for a three-taxon rooted tree with *n*_2_ being the youngest internal node and *n*_1_, the root node. This tree has fixed relative node ages *t*_2_ = 0.5 (age of *n*_2_) and *t*_1_ = 1.0 (age of *n*_1_). The corresponding absolute node ages are α*t*_2_ and α*t*_1_, where α is a scalar which takes values in [*u*/*t*_2_, 3]. *u* is the age of the fossil calibrating α*t*_2_, defining the younger bound for the corresponding node.

The two techniques aforementioned are not the only ones that can be used to define calibration constraints. In fact, any tree-generating model may extract some information from the available data about the marginal age of nodes used for calibrating the clock. In the following, I assess the relevance of tree-generating models for specifying calibration constraints by focusing on the particular case where genetic sequences are of infinite length and the correct models of sequence evolution and rate variation across edges are used for molecular dating. The relative node ages are then known with maximum precision. We observe a single fossil that helps us determine the scaling factor of all node ages, thereby allowing us to infer absolute (rather than relative) node ages. I will here consider the simple case where only three taxa are examined. *t*_1_ denotes the relative age of node *n*_1_, the MRCA of these three species, and *t*_2_ is the relative age of the calibrated node, *n*_2_ (see insert in Figure 5). Time grows backward with the present time set to zero. Finally, *u* is the age of the fossil. We therefore have 0 ≤ *u* ≤ α*t*_2_ ≤ α*t*_1_, where α is a scaling factor that defines the correspondence between relative and absolute node ages. Equation (5) in Stadler (2012) gives the expression for *p*_BD_(α*t*_2_|α*t*_1_, λ, μ), the probability density of α*t*_2_ given α*t*_1_ (and λ plus μ, the birth and death rates respectively, as well as the sampling fraction which we assume to be equal to 1.0 here) under the birth-death process with sampling, conditioned on the time of the MRCA of the three sampled species α*t*_1_. This expression serves as a basis to derive that of $p_{\text{BD}}\left(\alpha t_{2}|\alpha t_{1},t_{2}\geq u,\lambda ,\mu \right)=p_{\text{BD}}\left(\alpha t_{2}|\alpha t_{1},\lambda ,\mu \right)/\int_{u}^{\alpha t_{1}}p_{\text{BD}}\left(x|\alpha t_{1},\lambda ,\mu \right)\text{d}x$, i.e., the probability density of the age of node *n*_2_ conditioned on its value being greater than the age of the fossil used for the calibration, *u*.

Figure 5 focuses on the value of $p_{\text{BD}}\left(\alpha t_{2}|\alpha t_{1},\alpha t_{2}\geq u,\frac{\lambda }{\alpha },\frac{\mu }{\alpha }\right)\text{d}\left(\alpha t_{2}\right)$ (*y*-axis) as a function of α (*x*-axis). This expression corresponds to the conditional probability density of the age of *n*_2_ being equal to α*t*_2_, given that the age of *n*_1_ is equal to α*t*_1_ while the birth and death rates are equal to $\frac{\lambda }{\alpha }$ and $\frac{\mu }{\alpha }$, respectively. α is thus used here to control the pace at which the tree process unfolds. Values of this parameter greater than one therefore lead to a decrease in the rate at which birth and death of lineages take place, thereby pushing nodes in the phylogeny to be older. Values of the parameter smaller than one have the opposite effect. Note however that precise characterization of the relationship between α and the ages of *n*_1_ and *n*_2_ would deserve a more thorough examination.

When the fossil is found very near the tips of the tree (i.e., *u* = 0.01), the age distribution of node *n*_2_ is not influenced by the value of α, i.e., older ages and lower birth and death rates have the same probability as younger ages and higher rates. This observation is not surprising: parameters of the birth-death model are not identifiable from the relative node ages only, and multiplying the node heights by a given scaling factor while dividing the birth and death rates by the same factor leads to absolute node ages with the same probabilities. Increasing the value of *u*, i.e., considering older ages for the fossil, sees increasing amounts of information about absolute node ages. Indeed, there are substantial differences between probabilities of trees with young node ages (high probabilities, small values of α) compared to that of trees with older ages (lower probabilities, large values of α). Therefore, with older fossils, the probabilistic distribution of the absolute age of *n*_2_ is no longer flat as it is when *u* ≃ 0.0 and it becomes feasible to use the tree-generating process to define meaningful older bounds for calibration intervals. Results in Figure 5 therefore suggest that the calibration interval will be tighter for analyses that rely on older fossils compared to younger ones. A better understanding of this result may be obtained by considering how trees could be simulated under various constraints on the age of *n*_2_. The simplest approach would be to simulate under the standard birth-death process and then discard the generated trees where *n*_2_ is younger than *u*. It then becomes clear that the older *u*, the larger the proportion of discarded trees. The valid trees therefore represent a smaller proportion of all possible trees. In layman's terms, these trees therefore convey more information about the distribution of node ages compared to the case where no constraints apply.

The previous analysis focuses on the relationship between the age of the young bound for a calibration interval and the probabilistic distribution of the corresponding older bound. Yet, the applicability of these results depends on how accurate estimates of birth, death and, in the case of the FBD model, fossilization rates are (assuming, here again, complete sampling). In particular, the fossilization rate might be very difficult to estimate as it is influenced by a variety of factors. First, taphonomic phenomena are the source of major biases in the fossil record since organisms with hard body parts have a greater chance of being represented in this record. Moreover, the majority of fossils result from material deposited on the bottom of water bodies, thereby adding another source of bias shaping the fossil record. This heterogeneity in the fossil record is expected to show at large time scales. Smaller time scales, however, are expected to be less impacted by this phenomenon and the Poisson process arguably provides here a better description of the fossilization process.

**Figure 6:**
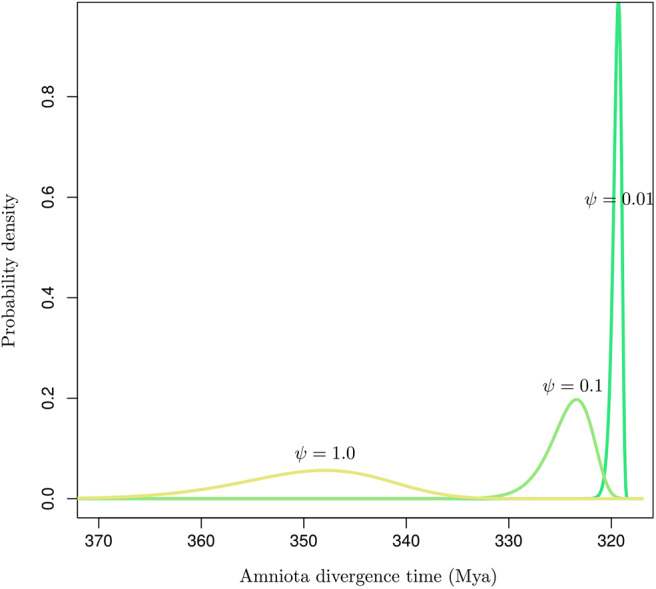
Distribution of the time of divergence of Amniota under the FBD model for various values of the fossilization rate (ψ). The birth and death rates were set to those given in Didier and Laurin (2020).

Inaccurate estimates of the fossilization rates may impact the inference of node ages substantially (Matschiner, 2019). Figure 6 shows the influence of the rate of fossilization on the probabilistic distribution of the age of a node that may then serve as a basis to calibrate the dating analysis. Didier and Laurin (2020) describe a method to derive the marginal distribution for the age of an internal node given the ages of all tips in a set of phylogenetic tree topologies. Using the same data set as in their article, comprising 109 dated extinct taxa covering Amniota and Diadectomorpha, I used the software DateFBD (available from https://github.com/gilles-didier/DateFBD) to infer the age of the Amniota clade. The estimation is conditioned here on a particular tree topology, in which Diadectomorpha is placed as outgroup. This topology is one of thousands of equally-most-parsimonious trees obtained from the analysis of a matrix of morphological characters (Didier and Laurin, 2020).

Although the three distributions are not conditioned here on having the same younger bound (i.e., by forcing the three densities to have the same 95% upper quantile for instance), substantial differences in their modes and the variances are observed depending on the value of the fossilization rate. Note however that the uncertainty around the fossilization rate estimated by Didier and Laurin (2020) is much smaller than the two orders of magnitude considered here. Their analysis focused on 109 data points corresponding to a rich collection of fossils. In situations where the fossil record is not as extensive as it is here, our results suggest that the conversion of node heights from molecular into calendar time units is highly sensitive to the fossilization rate estimate. Ages are indeed shifted toward older values for large values of that rate and vice versa. Accurate estimation of that rate is therefore paramount to the accurate inference of node ages. In practice, the sensitivity of the age estimates to the prior distribution on fossilization rate should thus be assessed on a systematic basis when using the FBD model, or any other tree-generating process, for molecular dating.

Stadler et al. (2018) recently proposed an extension of the original FBD model that accounts for multiple fossils per species and different modes of speciation. Although this work inches toward a more realistic modeling of the actual mechanisms generating the observations, more efforts are still needed in order to assess the robustness of the FBD model to deviations from the current hypotheses regarding the mechanisms of fossilization and the specifics of the data collection process. In particular, calibration information is generally provided by the oldest fossils in their respective clades instead of collections of randomly sampled fossils. In its current form, the FBD model does not take this information into account, which may result in substantial node age overestimation (Matschiner, 2019). The FBD also neglects information related to the absence of fossils in particular geological strata. In other words, this approach expects to find fossils in geological strata in which there is strong evidence *against* their presence. The absence of fossils is valuable information and node dating techniques provide a framework that helps accommodating for it in a simplified manner (see Marjanović and Laurin, 2007 for an example). Dealing with the absence of fossils in certain geological strata is not straightforward though. “Absence of evidence” should be distinguished from “evidence of absence.” Indeed, in the context of interest here, “absence of evidence” has to do with the way the sampling of geological strata was conducted. Models such as the birth-death skyline plot (Stadler et al., 2013) provide a relevant framework to accommodate for the variation in time of sampling intensity. “Evidence of absence” corresponds instead to the outcome of the data-generating process. Uneven preservation of fossils is one of the phenomena involved here. These processes need to be dealt with through adequate probabilistic modeling.

##### 3.2.2.3. Calibrating using marginal distributions

Molecular dating based on the FBD model can be considered a mechanistic approach as it relies on a model that mimics the actual process underlying splitting or extinction of lineages and fossilization events. The use of univariate probabilistic distributions to describe the age of certain clades has more to do with a phenomenological approach instead. Drummond et al. (2006) first introduced probabilistic distributions for node age calibration in the context of molecular dating using a fully Bayesian approach (but see Hedges and Kumar, 2004; Yang and Rannala, 2005 for an earlier introduction of this idea). They used normal and log-normal distributions but did not provide detailed explanations about the way the parameters of these distributions were selected from the analysis of the fossil record. Current approaches, available in BEAST (1 and 2) and MrBayes, implement standard statistical distributions, such as exponential, lognormal, or normal densities, with offset values set to the younger (i.e., minimum) age of the corresponding clade. mcmctree offers a selection of more sophisticated probabilistic distributions (see Yang and Rannala, 2005) but still relies on the same rationale. The older bound for each calibration is then defined by a variance parameter that controls for the probability that the age of the clade of interest is older than a given value, corresponding typically to the 95% upper quantile of the distribution. Hence, as opposed to the previous approach based on the FBD model, it is relatively straightforward here to define older bounds for each calibration interval. However, in its classical formulation, the “marginal distribution” approach does not account for uncertainty in the placement of the fossils in the phylogeny, which constitutes a serious limitation of that technique (but see Guindon, 2018 and below).

Drummond et al. (2006) account for the interaction between marginal priors^2^ and the joint distribution of the other node ages using an approach that does not respect fundamental rules of calculus with probabilities. Their approach results in multiple distributions (one defined by the marginal prior plus one derived from the tree-generating process) applying to the same node ages (Heled and Drummond, 2011; Warnock et al., 2015; Rannala, 2016). In Yang and Rannala (2005), calibrated and non-calibrated nodes are clearly separated in the derivation of a joint prior density of node ages that accommodates for fossil information. More specifically, let *T* = {*T*_1_, …, *T*_*n*−1_} denote the vector of all internal node ages *T*_1_ ≥ … ≥ *T*_*n*−1_ and Ψ the ranked tree topology with *n* tips. Both *T* and Ψ are random variables here. *t* and τ denote realizations of these random variables. *e* and *i* denote subsets of taxa and the corresponding time constraints respectively (R.V.: *E* and *I*). Each subset of taxa in *e* defines a clade and the corresponding element in *i* defines the time interval for the age of that clade. The joint density of the vector of node ages *t* and the tree topology τ, given calibration constraints *e*, *i*, is as follows:

(13) $$\begin{array}{l} p_{T,\Psi }(t,\tau |E=e,I=i)=\\ \;p_{T_{-c}}(t_{-c}|T_{c}=t_{c},\Psi =\tau )\times p_{T_{c}}(t_{c}|\Psi =\tau ,E=e,I=i)\\ \;\times \mathrm{Pr}(\Psi =\tau |E=e,I=i), \end{array}$$

where *T*_−*c*_ is the vector of node ages that are not subject to any calibration constraint. Yang and Rannala (2005) give an expression for the conditional density *p*_*T*_−*c*__(*t*_−*c*_|*t*_*c*_, τ) under the birth-death model with sampling. *p*_*T*_*c*__(*t*_*c*_|τ, *e, i*) is the joint density of the ages of all calibrated nodes. Yang and Rannala (2005) define this joint density as the product of the marginal “prior” densities used for calibration purposes. This definition is problematic since the joint density of calibrated ages is conditioned on the ranked tree topology. As a consequence, the calibrated ages cannot be considered independent from one another. In fact, when conditioning on a particular ranked tree, some combinations of node ages are not observable and the corresponding joint density should in fact be equal to zero. The probability of such “non-observable” outcomes depends on the parameters of the tree-generating process and cannot be simply ignored by the MCMC analyses used for Bayesian molecular dating. The discrepancy between the product of marginal calibration densities and the actual joint density that is used by these inference techniques is arguably the most obvious manifestation of the same issue (Heled and Drummond, 2011; Warnock et al., 2015). Rannala (2016) acknowledges this conundrum, only to reach the conclusion that “*the objective of preserving marginal calibrations is impossible to attain.”*

In Guindon (2018), I describe a new approach to node dating and provide a solution to the problem of uncertainty in the placement of fossil lineages in the tree. In this work, calibration constraints, *e* and *i*, are no longer considered as data. Instead, one acknowledges here that the actual data correspond to the fossils, noted as α (R.V.: *F*) and the calibration constraints then become parameters of the model, with inherent uncertainty. More specifically, the joint density of the time-tree and the calibration parameters given fossil data is now expressed as follows:

(14) $$\begin{array}{l} p_{T,\text{Ψ},E,I}\left(t,\tau ,e,i|F=\alpha \right)=p_{T,\text{Ψ}}\left(t,\tau |E=e,I=i\right)\\ \;\times p_{E,I}\left(e,i|F=\alpha \right), \end{array}$$

where *E* and *I* denote the random variables corresponding to subsets of taxa and the corresponding time intervals that, altogether, define calibration constraints. The term *p*_*E, I*_(*e, i*|*F* = α) serves as a basis to incorporate uncertainty in the calibration constraint due to ambiguity in interpreting the fossil data. In practice, experts may decide that a given fossil calibrates the age of the MRCA of species A and B with probability *p*, while the clade defined by species A, B, and C is calibrated by the same fossil with probability 1−*p*, thereby effectively accommodating for uncertainty related to fossil data. Moreover, the probabilistic distribution of the calibration constraints is not conditioned here on the tree topology. The combination of multiple calibration constraints therefore does not suffer from the issues mentioned above that are responsible for the discrepancy between “realized” distributions of calibrated node ages and the corresponding marginal priors. In other words, the marginal “priors” agree with their joint density as given by *p*_*E, I*_(*e, i*|α) (yet, the marginal distributions of calibrated node ages derived from *p*_*T*, Ψ_(*t*, τ, *e, i*|α) still disagree with these marginal priors, as expected from Equation 14).

Finally, the same approach may be extended in order to incorporate knowledge about the way fossils were collected. Equation (14) would then yield:

(15) $$\begin{array}{l} p_{T,\text{Ψ},E,I}\left(t,\tau ,e,i|F=\alpha ,S=s\right)=p_{T,\text{Ψ}}\left(t,\tau |E=e,I=i\right)\\ \;\times p_{E,I}\left(e,i|F=\alpha \right)\times p_{F}\left(\alpha |S=s\right), \end{array}$$

where the random variable *S* conveys information about the way sampling was conducted when collecting fossil data. For instance, the probability of observing a fossil of an ancient species that lived *X* million years ago would be equal to zero if sampling was conducted in geological strata corresponding to time intervals that do not include *X*. This term could also serve as a basis to incorporate geographical information in the analysis, translating the fact that some fossils are more likely to be found in particular regions and less in other areas.

#### 3.2.3. Model-Based Analysis of Fossil Data

The molecular dating methods presented above all rely on expert knowledge to determine, even approximately, where in the phylogeny fossil lineages should be placed. Yet, computational approaches can replace expert judgment here. Phylogenetic analyses of morphological data, including fossils, are most often conducted using parsimony. However, parametric methods like Bayesian inference, which use probabilistic models to describe the evolution of the morphological characters of which fossil data consist, can also be used. Such approaches offer the advantage that molecular data can be included in the same analysis (the “total-evidence” approach). Further, they can be combined with a tip-dating analysis to derive the joint posterior distribution of internal node ages from the combined probabilistic analysis of molecular and morphological data.

Pyron (2011) and Ronquist et al. (2012a) implemented this approach and analyzed concatenated alignments of molecular and morphological data. In this large matrix, molecular data is observed only for present-day sampled species (although ancient DNA is also a source of molecular data) while morphological data along with time information are available for both the sampled fossils and modern species. A phylogeny that incorporates fossils as *bona fide* taxa is then built from the analysis of this data. In this context, it is thus relatively straightforward to account for uncertainty in the placement of the fossil lineages. All the internal nodes on the path between a fossil tip and the root are constrained to be older than the age of the fossil. In theory, the time elapsed between fossil tips and the present could help define the rate of morphological evolution which would then serve as a basis to express all node ages in calendar time units.

Ronquist et al. (2012a) opted for an *ad hoc* approach where the rate of molecular evolution was first estimated using a node dating approach and then used as prior information in a subsequent dating analysis based on a total-evidence approach. Pyron (2011) also relied on a classical node dating approach to specify the distribution of the age of the root node. More recently, Gavryushkina et al. (2017) relied on a node dating approach too to calibrate the origin of the tree-generating process (thereby indirectly defining a marginal prior for the age of the root node), combined with a prior distribution on the rate of morphological evolution. Moreover, their approach rests on the FDB model as the tree-generating process, thereby using information from rates of birth, death and fossilization to further inform the absolute age of internal nodes (see section 3.2.2).

The three studies cited above are tip-dating analyses based on total evidence. They all relied on a classical node dating approach to derive absolute node ages from the combined analysis of molecular and fossil data. Although there is nothing wrong with mixing various approaches, the systematic reliance on node dating suggests that, at least in these three cases, morphological data alone may not have conveyed enough signal to infer reliable rates of morphological character evolution in practice, a necessary step for inferring absolute node ages in the absence of additional information for calibrating the clock. In fact, one may even wonder whether such a rate exists at all. Each morphological character having its own state space, one may indeed question whether it is meaningful to refer to an expected number of character changes per unit of calendar time (see Goloboff et al., 2018 for a discussion of this issue along with Goloboff et al., 2019). Furthermore, unlike for nucleotide or amino-acid characters, it is not always straightforward to define the alphabet of states for each morphological character (see e.g., Gavryushkina et al., 2017). dos Reis et al. (2016) also indicate that ascertainment biases due to the selection of parsimony-informative morphological characters from raw fossil data is difficult to deal with from a computational perspective and a proper correction, able to handle ambiguous alphabets of character states, is not implemented in any current software program for molecular dating.

## 4. Conclusions

Modeling the evolution of the rate of molecular evolution and accounting for fossil data are two challenging tasks that lie at the core of molecular dating techniques. Although much work has been done on these different aspects, in-depth exposition of the simplifications, the approximations, and the assumptions behind the proposed approaches helps gain a better understanding of their inherent strengths and limitations.

For instance, clearly separating the substitution rate trajectories that depict the fluctuations of the instantaneous substitution rates, from the average rates along edges of the phylogeny, leads to interesting observations. In particular, the “not-so-strict” clock model in which instantaneous rates vary while averages do not, could serve as a basis to revisit the clock hypothesis. At the very least, it constitutes an intermediate model between the strict and relaxed clock models that is worth considering. Furthermore, close examination of uncorrelated clock models reveals some of their shortcomings. The exponential clock model, in particular, has statistical properties that are not realistic from a biological perspective. More generally, uncorrelated clock models lead to stronger deviations from the strict clock constraint in trees with large numbers of tips compared to smaller trees, thereby revealing sampling-consistency issues that should be of concern. Autocorrelated clock models behave more sensibly altogether. Some of these models explicitly accommodate the variation of both instantaneous and average substitution rates without extra computational cost, making them superior to uncorrelated models from that point of view.

Taking into account fossil data in molecular dating experiments is another challenging statistical problem. The most recent techniques bet on an “all-modeling” approach that is hindered by a number of important limitations. In particular, unrealistic assumptions underlying the probabilistic models describing the evolution of selected morphological features should be of serious concern to total-evidence approaches. Assuming that fossils are “presence-only” data is also problematic. However, valuable information about the absence of some fossils in older geological strata is often available. The most recent inference techniques, including tip-dating and all approaches based on the fossilized-birth-death model, ignore this information, thereby enabling node age estimates that potentially contradict what is known from the fossil record. Node dating techniques rely on expert knowledge to define the position of fossils in the phylogeny plus the younger (and, oftentimes, the older) age bound(s) for the calibrated clades. Although expert knowledge involves subjectivity, which can be perceived as a weakness, one could argue that these approaches make better use of the available data for now. The future of molecular dating probably lies at the frontier between “all-expert” and “all-model” approaches whereby experts will provide prior information to plug into relevant statistical models for describing curated fossil data.

In any case, molecular dating will undoubtedly keep playing a crucial role in biology in the future. Our understanding of important phenomena such as species diversification or dispersal, population migration and demography, or the molecular signature resulting from environmental changes, depends on our ability to date past evolutionary events. The wealth of available techniques to perform this task provides a powerful set of tools to make progress in this direction. Yet, in-depth analysis of the mathematical and biological properties of the proposed new techniques, combined with rigorous and extensive assessments of their implementations, will be decisive to ensuring the soundness of our findings.

## Data Availability Statement

The raw data supporting the conclusions of this article will be made available by the authors, without undue reservation, to any qualified researcher.

## Author Contributions

The author confirms being the sole contributor of this work and has approved it for publication.

## Conflict of Interest

The author declares that the research was conducted in the absence of any commercial or financial relationships that could be construed as a potential conflict of interest.

## Footnotes

^1^https://fossilcalibrations.org/

^2^These marginal distributions are in fact derived from fossil data, and should thus not be considered prior densities *per se*, although most studies refer to them using this term based on the fact that marginal distributions are instantiated prior to observing genetic sequences.
