# Supplementary material for: Rates and Rocks: Strengths and Weaknesses of Molecular Dating Methods
Source: Front Genet. 2020 May 27;11:526. doi: 10.3389/fgene.2020.00526 (PMC7267027; doi:10.3389/fgene.2020.00526)
Supplement: Supplementary file 1 [file Data_Sheet_1.PDF]

Rates and rocks: strengths and weaknesses of molecular dating methods

SUPPLEMENTARY INFORMATION

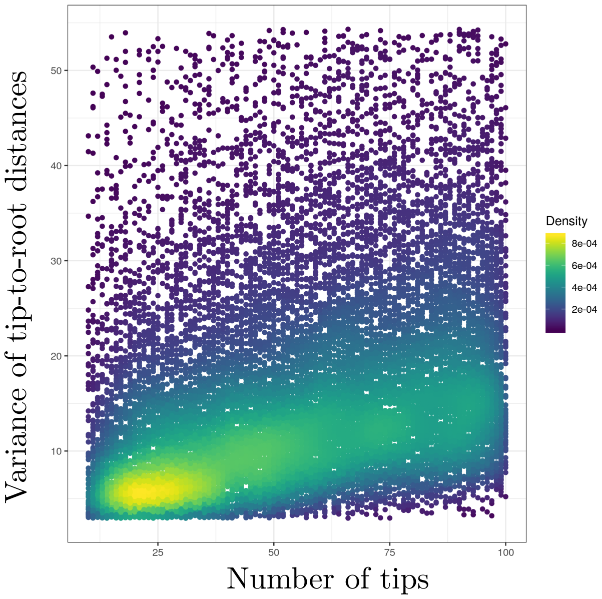

Figure S1. Uncorrelated clock models produce stronger deviations from the strict clock constraint in larger trees (gamma model). See caption of Figure 1 in the main text.

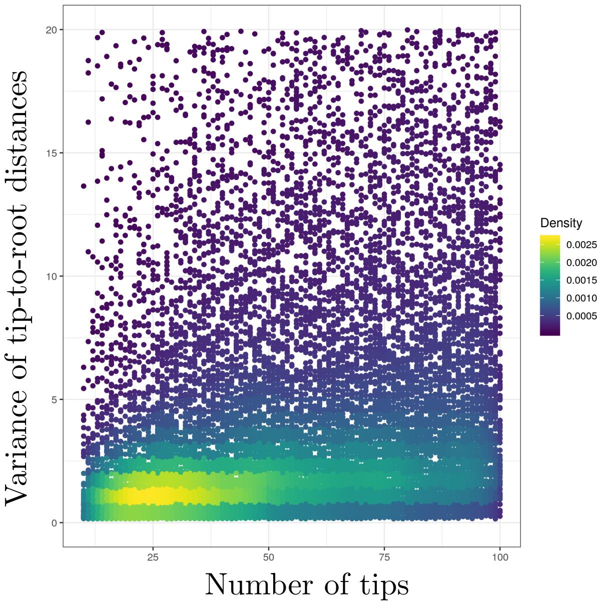

Figure S2. Autocorrelated clock models do not produce strong deviations from the strict clock constraint in larger trees. The autocorrelated model proposed by Kishino (2001) was used here.
